# Comparative feed management system in sheep fed different physical forms of ration containing *Ipomoea aquatica* on the performance, rumen characteristics, and chewing activity

**DOI:** 10.5455/javar.2023.j723

**Published:** 2023-12-31

**Authors:** Retno Adiwinarti, Edy Rianto, Endang Purbowati, Vita Restitrisnani, Agung Purnomoadi

**Affiliations:** Faculty of Animal and Agricultural Sciences, Universitas Diponegoro, Semarang, Indonesia

**Keywords:** Complete feeds, methane production, pelleted feeds, water spinach

## Abstract

**Objective:**

This study investigated the effectiveness of different physical forms of feed containing *Ipomoea aquatica* waste and concentrate feed on the rumen characteristics, chewing activity, and performance of sheep.

**Materials and Methods:**

Twenty-four rams (19.87 ± 2.19 kg) were arranged in a completely randomized design. Rams were fed dried *I. aquatica* waste and concentrate feed provided separately (RCF) (conventional feeding system), and total mixed ration consisted of mash complete feed (MCF), and pelleted complete feed (PCF). The data were analyzed using a one-way analysis of variance.

**Results:**

The dry matter intake (DMI), average daily gain, and feed conversion ratio of rams fed different physical forms of feed containing *I. aquatica* waste in their diet were similar between the treatments, ranging from 4.08% to 4.29% of body weight, 120–180 gm, and 6.32–9.17, respectively. Different physical feeds did not affect microbial synthesis in the rumen. Methane emissions per unit of production were similar between the groups. The PCF sheep ate faster (0.24 min/gm DMI) than the MCF sheep (0.38 min/gm DMI), but similar to the RCF sheep (0.31 min/gm DMI).

**Conclusion:**

Dried *I. aquatica* waste was useful as an alternative lamb feed roughage during the dry season. The pelleted mixed ration was more efficient than mash in increasing DMI. The dried *I. aquatica* waste was environmentally friendly for mitigating enteric methane emissions by sheep.

## Introduction

Water spinach (*Ipomoea aquatica*) is preferred for human consumption. In Indonesia, water spinach was widely planted in backyards to support food self-sufficiency during COVID-19, when people were forbidden to travel; therefore, water spinach waste was abundant. The water spinach waste was dried and can be used as potential feed for small ruminants during the dry season to reduce high feeding costs, as Gao et al. [1] stated that high feeding costs are one of the farming problems. Water spinach waste contains 10.65% of crude protein (CP), which is better than *Pennisetum purpureum* (CP: 6.70%) as a fiber source [2]. Maulana et al. [3] showed that local sheep fed water spinach *ad libitum* had average daily gain (ADG) values of about 118.19 gm (thin-tailed sheep) and 130.39 gm (“Garut” sheep). Therefore, *I. aquatica* can be used as a fiber source that is cheap and has better protein content for ruminants.

Nowadays, total mixed rations (TMRs) have replaced conventional feeding systems [4]. It can prevent feed sorting based on the palatability. Many researchers apply TMR in a variety of forms, such as mash [5] and pelleted TMR [6]. Li et al. [6] discovered that the better growth performance of lambs fed pelleted TMR was caused by an increase in feed intake. The physical form of feed will affect nutrient digestibility, as a pelleted-hay diet is easier to digest than loose hay [7]. Moreover, pelleted feed increased the ADG of sheep, feed efficiency, rumen bacteria [7], total digestible nutrients (TDNs), digestible crude fiber (CF), and growth performance of sheep [8]. The effect of the physical form of feed can be evaluated by observing chewing activity, ruminal pH, and feed intake [9].

Since *I. aquatica* waste has potential as a ruminant forage source during the dry season and TMR is practical to prevent the sortation of feed research about different physical forms of TMR containing *I. aquatica* is limited. Therefore, this study was designed to investigate the effect of different physical forms of feed containing *I. aquatica* waste as a fiber source on the performance, rumen characteristics, and chewing activity of fattening sheep.

## Materials and Methods

### Ethical approval

This research was held at the Faculty of Animal Agricultural Science Farm, Universitas Diponegoro (Indonesia). It has been approved by the Animal Ethics Committee of the Animal and Agricultural Science Faculty, Universitas Diponegoro, Indonesia (59-04/A-08/KEP-FPP).

### Animals, diets, experimental design, and management

About 24 rams [age: 10 months; body weight (BW): 19.87 ± 2.19 kg] were plotted in a completely randomized design and divided into three groups that were fed dried *I. aquatica* waste (45%) and concentrate feed (55%) provided separately (RCF) (conventional feeding system), and TMR consisted of mash complete feed (MCF) and pelleted complete feed (PCF) containing 45% of dried *I. aquatica* waste and 55% of concentrate feed. Dried *I. aquatica* waste was used as a roughage source. The concentrate consisted of cassava waste meal, soybean meal, pollard, molasses, and mixed minerals (Table 1).

The rams were reared for a 14-day adaptation, followed by 7 days for the preliminary treatment and 77 days for the treatment period. During the adaptation, all rams were injected with the anti-parasitic agent ivermectin, orally de-wormed with albendazole, and adapted to concentrate. All rams were fed three times daily and provided with 5% of their BW. Fresh water was provided *ad libitum*. Feed and water were controlled and added at 08:00, 12:00, and 16:00.

### Observed parameters

The parameters observed in this study were growth performance (ADG), feed conversion ratio (FCR), nutrient digestibility, nitrogen balance, N_2_O emission, rumen fermentation, methane emissions, microbial protein yield, and eating behaviors.

Feed digestibility was determined based on previous studies [10,11]. Seven days of total feces and urine collection were done during the ninth week of treatment. Rumen fluid was collected 3 h postfeeding [6] during the 10th week of treatment. It was analyzed for NH_3_–N concentration based on the Conway and O’Malley procedure [12] and volatile fatty acids (VFAs) by gas chromatography (Shimadzu GC-8). Methane (CH_4_) emissions were calculated based on Moss et al. [13]; that is, CH_4_ = 0.45 acetate – 0.275 propionate + 0.40 butyrate. The microbial *N* yield was calculated based on Chen and Gomes, cited by Khejornsart et al. [4]: *Y* = 0.84 *X* + (0.15 *W*^0.75^ *e*^−0.25*X*^), where *Y* is the excretion of purine derivatives in the urine (mmol/day); *W*^0.75^ is metabolic BW (kg), and *X* is absorbed microbial purines (mmol/day): *X* = (*Y* – 0.385 * *W*^0.75^)/0.85, and microbial *N* yield (gm N/day) = 0.727 * *X*. Eating and ruminating activities were recorded every 5-min interval for 48 h during the seventh week of the research, and lighting was provided at night during the research. Chewing time was calculated by adding the eating and ruminating times [14]. Mealtime was calculated by dividing the difference between eating time and total dry matter intake (DMI).

### Statistical analysis

Data were analyzed using a one-way analysis of variance. A *p*-value < 0.05 was considered significant, and treatment effects tended to be significant if 0.05 < *p* < 0.10 [6]. If a significant difference was detected, Duncan’s multiple-range test was used to compare means between the treatments.

**Table 1.** Nutrient contents of the feed ingredients.

Feed ingredients	DM	Ash	CP	EE	CF	NFE	TDN
	%	100% DM					%
Dried *I. aquatica* waste	86.38	17.30	10.11	0.59	27.16	44.84	48.67
Cassava waste meal	86.59	27.05	2.06	0.46	17.89	52.54	51.08
Soybean meal	88.50	10.75	32.09	3.15	5.47	48.54	79.43
Pollard	87.16	6.39	15.88	1.72	10.31	65.70	75.12
Molasses	77.33	9.13	3.67	0	1.14	86.06	82.51
Mixed minerals	98.54	95.53	0.17	1.08	0.09	3.13	21.85

DM: dry matter, CP: crude protein, EE: ether extract; CF: crude fiber; NFE: nitrogen-free extract; TDN: total digestible nutrients.

## Results

### Feed intake, feed conversion ratio, and growth performance

Composite samples of the rations are presented in Table 2. The DMI of all treatments was similar between the treatments (Table 3). However, the CP and CF intakes were significantly different (*p* < 0.05) between the treatments. In fact, the ADG and FCR between the treatments were also similar.

### Nutrient digestibility, nitrogen balance, and N2O emission

The nutrient digestibility of sheep fed the different physical form rations containing *I. aquatica* waste was similar, except for CP digestibility (Table 4).

Nitrogen (N) intake, fecal N (% N intake), N absorbed, N digestibility, and urinary N (% N intake) were significantly different (*p* < 0.05) between the treatments (Table 5). However, N retention between the treatments was similar.

Nitrogen emission and N_2_O emission (gm/day) were significantly different (*p* < 0.05) between the treatments. However, nitrogen emission and N_2_O emission per ADG were similar between the treatments (Table 6).

**Table 2.** Nutrient contents of the feed treatments.

Nutrients content	RCF		MCF and PCF
	Dried *I. aquatica* waste	Concentrate	
Dry matter (%)	86.38	87.20	86.83
Ash (%)	17.30	15.24	16.17
EE (%)	0.59	1.98	1.36
CF (%)	27.16	8.15	16.70
CP (%)	10.11	19.31	15.17
NFE (%)	44.84	55.32	50.61
TDN (%)	48.67	71.88	61.44

RCF = roughage (dried *I. aquatica* waste) and concentrate feed; MCF = mash complete feed; PCF = pelleted complete feed.

### Rumen fermentation and methane emissions

Rumen pH at 3 h postfeeding was similar (Table 7) between the treatments, with an average of 6.1. Total VFAs, acetate, butyrate, A/P ratio, and methane emissions were significantly different (*p* < 0.05) between the treatments. While propionate and NH_3_ contents were similar between the treatments.

### Microbial protein yield

The purine derivatives, rumen microbial N yield, and efficiency of microbial N production were similar between the treatments (Table 8).

### Chewing activity

Sheep in the PCF group spent chewing activities faster (*p* < 0.05) than others (Table 9). Their eating time was shorter than that of MCF sheep. The rumination time of TMR sheep was similar but faster than that of RCF sheep.

## Discussion

Different physical forms of ration affect nutrient intake. Sheep fed by conventional feeding systems (RCF) can sort the feed ingredients and eat more concentrate (62.58%) than dried *I. aquatica* waste (37.42%). It caused the ratio of roughage-concentrate intake of RCF sheep to be different from TMR (MCF and PCF) sheep. The roughage-concentrate ratio of TMR that was set up based on their nutrient requirements consisted of 55% concentrate and 45% dried *I. aquatica* waste. The higher concentrate intake of the RCF group affected CP and CF intake (Table 3), CP digestibility (Table 4), N balance (Table 5), and N and N_2_O emissions (Table 6). The CP intake of the RCF group was higher (*p* < 0.05) than that of the MCF group, but it was the same as the PCF group. The CF intake of the RCF sheep was lower (*p* < 0.05) than that of the PCF sheep, but it was similar (*p* > 0.05) to that of the MCF sheep. The intake of CP and CF by the TMR sheep was similar. The CP digestibility of the RCF sheep was higher than that of the TMR sheep, while the CP digestibility of the TMR sheep was similar. The CP digestibility of sheep fed a higher concentrate level is significantly higher than that of control sheep [15]. Unfortunately, the DMI of all treatments was the same, which was reflected in the similarity of the ADG (Table 3). The ADG was 150 gm and the average FCR was 7.71. It means that particle size did not affect total DMI or organic matter (OM) intake in sheep, as reported by previous researchers [16,17].

**Table 3.** Feed intake, BW, and FCR of sheep fed the different physical form rations containing *I. aquatica* waste.

Parameters	RCF	MCF	PCF	*p*-value
DMI (gm)	1,078.19 ± 154.26	992.28 ± 131.21	1,074.48 ± 181.57	0.48
DMI (%BW)	4.08 ± 0.43	4.17 ± 0.50	4.29 ± 0.25	0.58
CP intake (gm)	182.75^a^ ± 24.43	143.38^b^ ± 18.96	155.26^ab^ ± 26.24	0.01
CF intake (gm)	254.71^b^ ± 37.54	290.64^ab^ ± 38.43	314.71^a^ ± 53.18	0.04
Average daily gain (gm)	180.00 ± 50.00	120.00 ± 50.00	150.00 ± 50.00	0.11
Feed conversion ratio	6.32 ± 1.02	9.17 ± 3.4	7.63 ± 1.77	0.07

RCF = roughage (dried *I. aquatica* waste) and concentrate feed; MCF = mash complete feed; PCF = pelleted complete feed; DMI = dry matter intake; CP = crude protein; CF = crude fiber; BW = body weight.

^a,b^Different superscripts within a row indicate significance at *p* < 0.05.

**Table 4.** Nutrient digestibility of sheep fed the different physical form rations containing *I. aquatica* waste.

Parameters	RCF	MCF	PCF	*p*-value
DM digestibility (%)	66.03 ± 2.84	67.13 ± 1.72	65.33 ± 2.64	0.35
OM digestibility (%)	71.95 ± 1.86	73.29 ± 1.80	70.72 ± 3.84	0.18
CF digestibility (%)	51.20 ± 5.31	52.20 ± 7.09	59.73 ± 26.81	0.53
CP digestibility (%)	73.09^a^ ± 2.73	67.06^b^ ± 1.73	64.20^b^ ± 4.19	0.00
NFE digestibility (%)	83.84 ± 2.07	90.72 ± 6.12	81.33 ± 13.12	0.09
TDN (%)	60.44 ± 2.28	65.67 ± 1.77	61.91 ± 5.12	0.69

DM: dry matter; OM: organic matter; CF: crude fiber; CP: crude protein; NFE: nitrogen-free extract; TDN: total digestible nutrients; RCF = roughage (dried *I. aquatica* waste) and concentrate feed; MCF = mash complete feed; PCF = pelleted complete feed.

^a,b^Different superscripts within a row indicate significance at *p* < 0.05.

**Table 5.** Nitrogen balance in sheep fed the different physical form rations containing *I. aquatica* waste.

Parameters	RCF	MCF	PCF	*p*-value
N intake (gm/day)	30.84^a^ ± 5.20	23.76^b^ ± 3.40	28.36^ab^ ± 5.12	0.02
Fecal N (gm/day)	8.37^ab^ ± 1.86	7.84^b^ ± 1.27	10.14^a^ ± 2.03	0.04
Fecal N (% N intake)	26.9^c^ ± 2.73	32.94^b^ ± 1.73	35.80^ab^ ± 4.19	0.00
N digestibility (%)	73.09^a^ ± 2.73	67.06^b^ ± 1.73	64.20^bc^ ± 4.19	0.00
Urinary N (gm/gm)	10.4^a^ ± 2.53	5.88^b^ ± 1.37	7.44^b^ ± 1.58	0.00
Urinary N (% N intake)	33.61^a^ ± 5.00	24.73^b^ ± 4.77	26.21^b^ ± 2.63	0.00
N retention (gm/day)	12.07 ± 2.39	10.05 ± 1.85	10.78 ± 2.63	0.23
N retention (% N intake)	39.48 ± 6.29	42.33 ± 5.39	37.99 ± 5.65	0.33

RCF = roughage (dried *I. aquatica* waste) and concentrate feed; MCF = mash complete feed; PCF = pelleted complete feed.

^a,b,c^Different superscripts within a row indicate significance at *p* < 0.05.

The ADGs in this study (Table 3) were higher than those in previous studies, such as 31.25–134.38 [18] and 54.4–92.5 gm/day [19]. The FCR of the RCF (6.32) and PCF (7.63) lambs was better than that of feedlot lambs (8.61–9.07), as reported by Saldanha et al. [20]. Matar et al. [16] reported FCRs of 7.12–8.05 using a pelleted TMR.

Based on Table 5, the positive N balance indicated that the sheep ate adequate protein [11]. The highest N absorbed by the RCF sheep was affected by a high N intake. It was caused by the DMI of RCF sheep containing more concentrate (Table 3) that was rich in protein content (Table 2). The higher urinary N excretion in the RCF group was caused by higher N digestibility (Table 5), which produced more ammonia (NH_3_) in the rumen (Table 7) and was metabolized as urea in the urine [11]. Therefore, N_2_O emissions from RCF sheep were higher than those from MCF sheep. However, no significant difference was detected between the treatments (Table 6) if nitrogen emission and N_2_O emission were counted per unit of production.

In this study, the rumen pH of 6.1 (Table 7) was favorable for rumen microbial protein synthesis. Previous studies have reported that a rumen pH between 6.2 and 7.0 was the best for fiber and starch digestion [18,21]. Total VFAs of the RCF sheep were higher than those of the PCF sheep, indicating a higher rumen fermentation rate in RCF sheep, as reported by Lu et al. [22]. In this study, the increase in rumen VFAs was generated by protein fermentation (Table 5). The amount of N absorbed was highest in the RCF group. Propionate in the RCF group tended to be the highest because the DMI of the concentrate was higher than in the other treatments. The propionate concentration accelerated because of the increase in the concentrate feed [18].

**Table 6.** Nitrogen emission and N_2_O emission from sheep fed the different physical form rations containing *I. aquatica* waste.

Parameters	RCF	MCF	PCF	*p*-value
N emission (gm/day)	18.78^a^ ± 4.12	13.71^b^ ± 2.33	17.58^ab^ ± 3.44	0.018
N emission (% N intake)	60.52 ± 6.29	57.67 ± 5.39	62.01 ± 5.65	0.333
N emission gm/kg ADG	108.79 ± 20.44	125.63 ± 45.77	125.07 ± 32.45	0.550
N_2_O emission (gm/day)	0.38^a^ ± 0.08	0.27^b^ ± 0.05	0.35^ab^ ± 0.07	0.019
N_2_O emission gm/kg ADG	2.18 ± 0.41	2.51 ± 0.92	2.50 ± 0.65	0.550

RCF = roughage (dried *I. aquatica* waste) and concentrate feed; MCF = mash complete feed; PCF = pelleted complete feed.

^a,b^different superscripts within a row indicate significance at *p* < 0.05.

**Table 7.** Rumen fermentation of sheep fed the different physical form rations containing *I. aquatica* waste.

Parameters	RCF	MCF	PCF	*p*-value
Rumen pH	6.3 ± 0.6	6.1 ± 0.9	5.8 ± 0.5	0.40
VFA (m Mol)	67.19^a^ ± 12.89	51.95^ab^ ± 27.43	38.75^b^ ± 13.27	0.03
Acetate (m Mol)	51.13^a^ ± 10.12	38.98^ab^ ± 19.87	27.15^b^ ± 9.18	0.01
Propionate (m Mol)	12.79 ± 4.08	8.27 ± 5.13	8.47 ± 3.24	0.08
Butyrate (m Mol)	6.11^a^ ± 2.02	4.71^ab^ ± 3.03	3.12^b^ ± 1.43	0.05
A/P ratio	4.21^ab^ ± 0.89	4.99^a^ ± 0.87	3.30^b^ ± 0.81	0.00
CH4 (mol/100 mol)	21.93^a^ ± 4.17	17.15^ab^ ± 8.85	11.14^b^ ± 3.96	0.01
Ruminal NH_3_ (mg/100 ml)	20.25 ± 2.42	18.76 ± 3.02	19.82 ± 2.63	0.53

RCF = roughage (dried *I. aquatica* waste) and concentrate feed; MCF = mash complete feed; PCF = pelleted complete feed.

CH_4_ emissions were calculated based on Moss et al. [13]; that is, CH_4_ = 0.45 acetate – 0.275 propionate + 0.40 butyrate.

^a,b^Different superscripts within a row indicate significance at *p* < 0.05.

The average ruminal NH_3_–N in this study (19.61 mg/100 ml) was higher than those in Olafadehan and Okunade [10] research (15.60–18.00 mg/100 ml). However, it was lower than Wang et al. [23], who reported 24.76–28.84 mg/100 ml. These differences were caused by differences in N intake and N digestibility (%), as reported by Olafadehan and Okunade [10]. Getahun et al. [24] stated that dietary protein is the main source of NH_3_ production in the rumen. Ammonia, as a nitrogen source, is utilized by bacteria to produce amino acids and peptides required for growth [24]. The minimum ruminal NH_3_ for optimal fermentation by microbes is >15.00 mg/100 ml [10].

Table 7 shows that methane emissions from the RCF sheep were higher than those from the PCF sheep. However, the methane emissions per unit of production were similar between the groups. Methane production in this study (11.14–21.93 mol/100 mol) was lower than that reported by Okunade and Olafadehan [11] at 20.00–24.00 mol/100 mol. Therefore, *I. aquatica* waste can be used as an alternative feed during the dry season. This is an environmentally friendly feed that mitigates enteric methane emissions from lambs for a sustainable environment.

Based on Table 8, the different physical feeds did not affect microbial synthesis in the rumen. The average microbial N yield in this study was 1.84 gm/day. This finding agrees with Vidya et al. [25] that particle size did not affect microbial N supply because the ruminal pH, DMI, and ruminal NH_3_ were the same. Factors affecting rumen microbial production are ruminal pH, feed intake, and ammonia from degraded protein [26]. The ruminal NH_3_–N concentrations in this study were in the optimal range (15–20 mg/100 ml) [11].

**Table 8.** Urine, purine derivatives, microbial N yield, and efficiency of microbial N production of sheep fed the different physical form rations containing *I. aquatica* waste.

Parameters	RCF	MCF	PCF	*p*-value
Purine derivatives (mmol/day)	0.93 ± 0.26	0.74 ± 0.19	0.74 ± 0.20	0.15
Microbial N yield (gm N/day)	2.49 ± 2.15	1.74 ± 1.2	1.28 ± 1.02	0.35
Efficiency of microbial N production				
gm/gm digested N	0.12 ± 0.13	0.10 ± 0.06	0.08 ± 0.07	0.65
gm/gm N intake	0.09 ± 0.10	0.07 ± 0.04	0.05 ± 0.04	0.48

RCF = roughage (dried *I. aquatica* waste) and concentrate feed; MCF = mash complete feed; PCF = pelleted complete feed.

**Table 9.** Chewing activity of sheep fed the different physical form rations containing *I. aquatica* waste.

Parameters	RCF	MCF	PCF	*p*-value
Eating (min/day)	327.86^ab^ ± 87.60	370.36^a^ ± 75.59	254.64^b^ ± 59.89	0.03
Rumination (min/day)	316.43^a^ ± 76.81	120.71^b^ ± 34.90	85.71^b^ ± 35.23	0.00
Chewing (min/day)	644.29^a^ ± 138.61	491.07^b^ ± 67.36	340.36^c^ ± 68.44	0.00
DMI (gm/day)	1066.59 ± 199.05	997.73 ± 160.49	1103.32 ± 351.46	0.73
Mealtime (min/gm DMI)	0.31^ab^ ± 0.07	0.38^a^ ± 0.09	0.24^b^ ± 0.06	0.01

RCF = roughage (dried *I. aquatica* waste) and concentrate feed; MCF = mash complete feed; PCF = pelleted complete feed.

^a,b,c^Different superscripts within a row indicate significance at *p* < 0.05.

Chewing activities of sheep fed TMR were faster (*p <* 0.05) than those of RCF sheep (Table 9). This was because the particle size of the complete feed ration, both in the form of mash (MCF) and pellet (PCF), was smaller than that of the RCF ration containing dried *I. aquatica* waste chopped coarsely. Therefore, the rumination times of MCF and PCF sheep were shorter than those of RCF sheep. Banakar et al. [9] stated that chewing activity can be faster if forages are ground to a smaller particle size. The PCF sheep ate faster (min/gm DMI) than MCF sheep, but similar to RCF sheep. This indicated that pelleted feed (PCF) was easier to eat than mash feed (MCF). In this case, the mash form is more difficult to get into the mouth; therefore, the MCF mealtime took longer than the PCF mealtime. It is concluded that the pelleted form is more efficient than the mash form to increase DMI. In addition, mash feed was easier to spill out of the feeder than pelleted feed. Li et al. [6] stated that pelleted feed can reduce the amount of feed waste. Chewing activities (min/gm of DM) in this study (mealtime in Table 9) were less than those reported by Daza et al. [27], which were about 0.66–1.02 min/gm of DM. It indicated that the ration containing *I. aquatica* waste in this study was eaten faster than the ration of Daza et al. [27] research containing ground licuri (*Syagrus coronate*).

This study showed that the conventional feeding management system (RCF) allows sheep to choose the feed having better palatability (concentrate), so the aim to utilize dried *I. aquatica* waste in the ration was not achieved. It is suggested that a TMR (MCF and PCF) is the best way to mix unpalatable feedstuffs so that sheep cannot get rid of the dried *I. aquatica* waste. Therefore, the utilization of dried *I. aquatica* waste to achieve more economic benefits and a sustainable meat product during the dry season can be achieved.

## Conclusion

It can be concluded that the different physical forms of feed containing *I. aquatica* waste in the diet did not affect the productivity of the sheep with an ADG of 120–180 gm and an FCR between 6.32 and 9.17. Dried *I. aquatica* waste was useful as an alternative lamb feed during the dry season. The pelleted mixed ration was more efficient than mash to increase DMI. The dried *I. aquatica* waste was environmentally friendly for mitigating enteric methane emissions by sheep.

## List of abbreviations

ADG, average daily gain; BW, body weight; CF, crude fiber; CP, crude protein; DMI, dry matter intake; EE, ether extract; FCR, feed conversion ratio; MCF, mash complete feed; NFE, nitrogen extract; OM, organic matter; PCF, pelleted complete feed; RCF, dried *I. aquatica* waste (45%) and concentrate feed (55%) provided separately; TDN, total digestible nutrients.
